# Seabird’s cry: repertoire and vocal expression of contextual valence in the little auk (*Alle alle*)

**DOI:** 10.1038/s41598-023-35857-3

**Published:** 2023-05-27

**Authors:** Anna N. Osiecka, Elodie F. Briefer, Dorota Kidawa, Katarzyna Wojczulanis-Jakubas

**Affiliations:** 1 Department of Vertebrate Ecology and Zoology, Faculty of Biology, University of Gdańsk, 80-308, Gdańsk, Poland; 2 Behavioural Ecology Group, Section for Ecology and Evolution, Department of Biology, University of Copenhagen, 2100 Copenhagen, Denmark

**Keywords:** Behavioural ecology, Animal behaviour

## Abstract

Many seabird species breed in colonies counting up to hundreds of thousands of individuals. Life in such crowded colonies might require special coding–decoding systems to reliably convey information through acoustic cues. This can include, for example, developing complex vocal repertoires and adjusting the properties of their vocal signals to communicate behavioural contexts, and thus regulate social interactions with their conspecifics. We studied vocalisations produced by the little auk (*Alle alle*)—a highly vocal, colonial seabird—over mating and incubation periods on the SW coast of Svalbard. Using passive acoustic recordings registered in a breeding colony, we extracted eight vocalisation types: *single call, clucking*, *classic call*, *low trill*, *short call*, *short-trill*, *terror*, and *handling* vocalisation. Calls were grouped by production context (based on the typically associated behaviour), to which a valence (positive vs negative) was later attributed, when possible, according to fitness threats, i.e. predator or human presence (negative) and promoters, i.e. interaction with a partner (positive). The effect of the putative valence on eight selected frequency and duration variables was then investigated. The putative contextual valence significantly affected the acoustic properties of the calls. Calls assigned positive valence had higher fundamental frequency and spectral centre of gravity as well as shorter sound duration than these assigned negative valence. These results indicate that the little auk’s vocal communication system may facilitate expression of complex behavioural contexts, and seems to include vocal plasticity within vocalisation types—however, more data are necessary to better understand this effect and possible interplays of other factors.

## Introduction

Vocal communication is fundamental for many animal species^1^. Acoustic cues can carry important information about the individual, e.g., the callers’ identity^2,3^, sex^4^, size or quality^5,6^, but also about the behavioural context^7,8^ and affective state^9^. Because of this, vocal signals facilitate many social interactions^10^, and can become particularly complex in socially cohesive species^11^.

For animals living in large aggregations, using acoustic cues may become problematic to use due to noise and density of neighbours. Therefore, life in colonies requires a species to adjust their coding–decoding system to communicate efficiently. This may lead to developing, for example, complex vocal repertoires (i.e. many different call types) or complex acoustic features (e.g. segregation of information in distinct, independent features) for more efficient communication^12,13^, as well as temporal and frequency adjustments allowing to convey fine behavioural contexts^9,14–16^. All this can result in an increased vocal variability.

One of the drivers behind vocal complexity can be the need to accurately convey different affective contexts—this is particularly crucial for social animals, aiding in areas such as e.g. conflict de-escalation, predator avoidance, and food location. Affective states, or emotions, are short-term states elicited in response to specific stimuli of importance for the organism, and associated with neuro-physiological, behavioural and cognitive changes^17^. According to the ‘two-dimensional’ approach, they can be characterised by their arousal (bodily activation) and valence (positive or negative, i.e. intrinsic pleasantness or unpleasantness^18,19^). Their function is to guide adaptive behaviour to promote survival: positive states are triggered by stimuli that enhance fitness and usually result in an approach towards the stimulus, while negative states are elicited by stimuli that threaten fitness and hence often induce avoidance of the stimulus^18^.

Studies of affective responses in non-human animals require taking their perspective and deciding on robust, measurable parameters^20^. One promising indicator of affective valence are vocalisations^9,9–22^—depending on the animal’s state, its calls’ acoustic properties may change^9^, e.g. in sound duration and fundamental frequency variation^9,23^. Importantly, since vocal expressions of emotions often carry crucial information about the environment or social interactions, conspecifics can perceive them^24^ and respond to them^14^. Because of the relative difficulty in objectively assessing emotional responses of non-human animals and creating controlled conditions in the wild, this topic remains relatively understudied in non-captive animals.

Most seabirds breed in large colonies, and many are known for their complex social networks^25–27^ and high vocal activity. Nevertheless, vocal communication in this group is still poorly understood and notably overlooked in the scientific literature that has focused mostly on passerines. This knowledge gap is mostly due to the difficulty to study seabirds (e.g. time spent at sea, noisy environments, and difficult access), and inquiries into their acoustic behaviour is still typically limited to partial repertoire descriptions. However, recent findings show that calls of some of these species can be individually and contextually specific^7,16,28^, indicating exciting new areas of seabird acoustic communication.

The little auk (*Alle alle*) is a long-lived, colonial seabird^29^ with a strong nest- and partner fidelity^30,31^, suggesting complex and cohesive social networks. Being the most numerous Arctic seabird species^31,32^ living in dense, populous colonies^29^, it is also relatively easy to access, making it a great model for behavioural studies^31^. Even though the little auk is a very vocal species, its acoustic behaviour remains undescribed, with only some brief observations available^33^.

Here, we examine calls produced by little auks in a breeding colony in SW Svalbard. Our main objective was to provide a detailed quantitative and qualitative description of the vocal repertoire of adults, to set a reference framework for future studies. Further, we investigated whether and how affective states may affect the acoustic properties of these calls.

## Results

### Repertoire

We visually identified, based on their spectrogram and assigned production context (Table 1), eight distinct vocalisation types produced over the mating and incubation periods: *single call, clucking, low trill, short call, short-trill, single call, terror*, and *handling* (Tables 1, 2). Further tests showed that these calls differed significantly in their acoustic parameters (Permutation test: correct classification based on a discrimination function analysis = 61.7%; correct classification by chance = 12.5%; p < 0.001).

**Table 1:** Little auk vocalisations produced by adults over mating and incubation periods.

Vocalisation type	Most common production context	Assigned valence	Example	Description
Single call (single call)	Flying alone or in flock, sitting alone in the colony	Unknown		Produced as a single vocalisation. Rarely observed
Clucking (aggressive call/clucking call)	Vocalising and posturing with a partner, pre-, post- or without copulation, as well as during other encounters and prolonged stays in the colony with the social partner	Positive		Produced in vocalisation bouts: spectrograms show a single cluck (left) and a vocal exchange between breeding partners (right). Observed often
Classic call (trilling call)	Flying alone or in flock, also during predator escape, in and outside nest	Unknown		The longest call of the species, composed as a single call made of a series of three syllable types. Observed often
Terror	Flying in flock when a predator is present on the ground	Negative		Produced as a series of 2–6 identical syllables. Observed often within the behavioural context, often at the same time by many individuals
Handling	Vocalisation during human handling	Negative		Produced as a single call. Observed rarely and only during the artificial context of human handling
Low trill (snarling call)	In or in front of own nest when another bird is present	Likely negative		Observed often, sometimes in a bout with the *short* and *short with trill* calls
Short call	In or in front of own nest when another bird is present	Likely negative		Observed often, produced as a single call or in a bout with the *low trill* and *short with trill* calls
Short-trill	In or in front of own nest when another bird is present	Likely negative		Somewhat a combination of the *short* and *low trill* calls and often produced in a bout with these. Observed often

Spectrograms plotted using the *seewave* package (Sueur et al. 2008^34^). If calls were described previously by Ferdinand^33^, names used therein are given in brackets. See also the summary statistics for acoustic parameters of the calls in Table 2, and Data availability statement for call samples.

**Table 2:** Values (mean ± SD) of acoustic parameters for the little auk call types.

Acoustic parameter	Call type							
	Single	Clucking (+)	Classic	Terror (−)	Handling (−)	Low trill	Short	Short-trill
*f0* Min (Hz)	657.15 ± 97.87	646.67 ± 58.99	632.45 ± 60.50	657.98 ± 73.97	685.62 ± 108.49	621.99 ± 84.97	657.49 ± 72.74	601.90 ± 63.01
*f0* Max (Hz)	808.76 ± 177.02	809.65 ± 95.53	1209.48 ± 154.24	766.58 ± 72.57	749.48 ± 135.66	915.47 ± 133.68	815.05 ± 78.40	934.72 ± 104.98
*f0* Mean (Hz)	759.09 ± 150.86	751.51 ± 80.18	912.61 ± 63.60	721.23 ± 65.30	717.65 ± 118.14	808.71 ± 108.99	735.81 ± 65.08	808.49 ± 77.38
Q25% (Hz)	676.43 ± 477.10	1334.59 ± 498.92	1504.70 ± 588.35	74.84 ± 271.09	377.78 ± 297.60	802.11 ± 344.17	708.10 ± 154.63	769.52 ± 286.84
Q50% (Hz)	1375.92 ± 846.78	2073.89 ± 410.24	2634.73 ± 1499.41	794.57 ± 2503.71	770.45 ± 385.00	1927.92 ± 1508.20	1390.37 ± 938.34	1589.59 ± 1303.54
Q75% (Hz)	3939.74 ± 4299.23	2718.37 ± 442.53	5278.29 ± 4903.84	1535.94 ± 4346.71	1457.16 ± 451.01	6989.95 ± 5834.44	5473.14 ± 4549.35	4417.26 ± 4727.89
Duration (s)	0.11 ± 0.05	0.09 ± 0.02	2.74 ± 0.44	0.33 ± 0.13	0.11 ± 0.10	1.08 ± 0.84	0.23 ± 0.15	0.75 ± 0.38
AM rate (s^−1^)	28.64 ± 10.71	26.28 ± 12.44	39.11 ± 8.08	42.21 ± 9.40	24.62 ± 11.09	47.98 ± 4.90	37.08 ± 12.17	45.40 ± 4.87
*f*M rate (s^−1^)	4.78 ± 3.67	5.53 ± 1.00	5.65 ± 0.76	8.76 ± 2.56	5.13 ± 4.18	4.37 ± 2.05	2.77 ± 2.51	4.05 ± 1.72

Positive and negative emotional valence are denoted as (+) and (−), respectively.

### Effects of affective valence on the acoustic parameters

MANOVA testing (with call types as a control fixed factor) of calls attributed to contexts of clear positive and negative valence revealed that the assigned affective valence had a significant effect over the call structure (F = 48.93; p < 0.001; Supplementary Table 1). Further linear models revealed that calls assigned to a positive valence (i.e. *clucking* calls exchanged between partners) had significantly higher values of acoustic parameters such as Max *f0*, Mean *f0*, *f0* Range, Q50%, *f0* Abs Slope, and *f0* var, and significantly lower values of acoustic parameters such as End *f0* as well as shorter sound duration than those assigned to a negative valence (i.e. *terror* and *handling* calls produced in response to threat; Table 3; Supplementary Figs. 1–8; see Supplementary Table 2 for parameters’ definitions).

**Table 3:** Model results: linear models for negative (intercept) and positive emotional valence.

		Predictors		R2/R2 adjusted
		Negative valence (Intercept)	Positive valence	
Max *f0* (Hz)	Estimates	758.03	51.62	0.053/0.042
	95% CI	731.30–784.76	5.32 to 97.92	
	p	< 0.001	0.029	
Mean *f0* (Hz)	Estimates	719.44	32.07	0.028/0.017
	95% CI	696.31–742.57	− 7.99 to 72.12	
	p	< 0.001	0.115	
Range *f0* (Hz)	Estimates	86.23	76.75	0.259/0.251
	95% CI	70.35–102.11	49.25 to 104.26	
	p	< 0.001	< 0.001	
Q50% (Hz)	Estimates	782.51	1291.38	0.149/0.139
	95% CI	404.56–1160.46	636.75 to 1946.01	
	p	< 0.001	< 0.001	
*f0* abs slope	Estimates	1014.81	2005.70	0.566/0.561
	95% CI	799.83–12,229.78	1633.35 to 2378.05	
	p	< 0.001	< 0.001	
*f0* Var (Hz/s)	Estimates	764.25	1687.60	0.618/0.614
	95% CI	602.09–926.42	1406.73 to 1968.47	
	p	< 0.001	< 0.001	
End *f0* (Hz)	Estimates	699.09	− 47.36	0.065/0.055
	95% CI	677.16–721.01	− 85.34 to − 9.38	
	p	< 0.001	0.015	
Sound duration (s)	Estimates	0.22	− 0.13	0.188/0.179
	95% CI	0.19–0.26	− 0.19 to − 0.07	
	p	< 0.001	< 0.001	

No. of observations = 90 for all parameters.

Calls of unknown or uncertain valence showed the largest variance for most of the measured parameters (Supplementary Figs. 1–8).

## Discussion

The complexity of vocal communication can be predicted to be high in socially cohesive^11^ and colonial species^18,35^, in terms of the size of a species’ vocal repertoire, but also of the fine information coding (e.g. context, internal state) within call types. Here, we described eight vocalisation types with distinct acoustic structures produced by adult little auks over the mating and incubation periods at the colony site.

Additionally, we tested how these calls might be affected by the animal’s putative affective states. Based on their production context, we compared call types associated with putative positive (interactions with a social partner) and negative (response to a predator or human) valence, and found valence-specific changes. This shows that the species uses complex vocal communication, capable of conveying fine contextual information useful in social interactions.

### Vocal repertoire

We observed eight acoustically distinct call types produced by the little auks in various contexts over the mating and incubation periods. Overall, little auk vocalisations are high frequency calls, with a clear harmonic structure, typical for species living in open habitats^36^, such as the high-Arctic tundra. Such harmonic calls facilitate both signal transmission and location of the caller^37^, which may be beneficial at a colony scale, e.g. for locating alarm calls and social partners in an acoustically crowded environment.

Little auks vocalise in different behavioural contexts, yet most calls are used in social exchanges. Half of the described call types are produced during direct interactions with conspecifics: *clucking* is most commonly uttered in a vocal dyad with a social partner, and *low trill*, *short call*, and *short-trill* are used when another bird is present and/or engages in a vocal exchange. Two call types (*terror* and *handling*) are produced only when a predator or threat (such as a human observer) is present nearby, likely serving as a warning to the colony.

While some calls are highly context-specific (e.g. *clucking* or *handling*), others may be used in various situations. The most striking example is the *classic* call—a sound any visitor to the areas inhabited by the little auk will instantly recognise as its “signature”, and which in some languages (such as Polish and Norwegian) served as the inspiration for the species’ name and/or its breeding sites. This vocalisation is used by birds returning to the breeding colonies after wintering, foraging trips, and during predator escape—in all cases, huge flocks vocalise together, resulting in a cacophony of voices. However, the *classic* call can also be heard from single birds flying or sitting outside or inside their nests. The acoustic properties of this call type are quite variable, suggesting vocal plasticity in adjusting a call type to convey a finer message.

It has previously been suggested that colonial alcids may have larger repertoire sizes than solitary-nesting species^12^. While to date no complete repertoire descriptions (i.e. covering at-sea, migration, and wintering vocalisations) exist for any of the alcid species, the little auk seems to have a larger repertoire than those described so far for most other alcids (i.e. auklets, puffins, and murres, typically with up to 5 call types described per species^12,38,39^), and much larger than the repertoire of the solitary Kittliz’s murrelet *(Brachyramphus brevirostris*), with only two call types described^40^. Taking into account that this description only focuses on the vocalisations of adult birds over a selected period (mating and incubation), and other calls are likely emitted in contact with the chick and at sea, the complexity of little auk’s repertoire indicates complex vocal signalling at the colony level.

### Behavioural and affective context

Previous studies on vocal expression in seabirds showed that production context impact the acoustic properties of their calls^15,16^. Similarly, little auk vocalisations differ significantly across behavioural, but also assumed affective contexts. This is the first time that the impact of the putative valence on acoustic parameters has been investigated in a seabird. The acoustic variables contributing most to the observed variation between calls were related to the fundamental frequency (*f0*), modulations of which are commonly associated with affective states^9,41^. Calls produced during interactions with a partner (i.e., increasing fitness) were significantly shorter than these uttered during vocal exchanges with other birds or associated with negative contexts (e.g., handling by a human or in presence of a predator). This is in line with vocal expression patterns in previously described species^9,21^. At the same time, while positive vocalisations generally tend to have lower frequency attributes^9,21^, these parameters were higher (e.g., higher fundamental frequency and frequency modulation) for little auks. While calls produced in multiple behavioural contexts (i.e. *single* and *classic* calls) seem to show a larger variance in acoustic parameters, potentially indicating vocal plasticity within a call type, the design of this study is too general to make such conclusions, but it certainly calls for a more thorough investigation in the future.

In the absence of other measures of emotions (e.g. behavioural or cognitive), the putative valence of the contexts was assessed based on whether the context promotes (positive valence) or threatens (negative valence) the fitness of the animal^17,19^. Our results are thus limited to the assumptions that affective states have indeed evolved to promote fitness (survival and reproduction), and that positive and negative states are triggered in such contexts (e.g. interactions with a partner were assigned positive valence, vocalising in the presence of a predator or during handling by a human were assigned negative valence; see Table 1. This also additionally limits the available dataset, since in this analysis we could only include calls that could be assigned to a positive or negative state with high probability, and thus calls of unknown or uncertain (i.e., *often negative*) valence had to be discarded. In addition, we did not have access to the second main dimension of emotions, which is the arousal (bodily activation) of the vocalising animals^42^. Some of the effect we found might thus be related to arousal more than valence—e.g., high arousal during vocalisation bouts related to copulation might result in increased frequencies and frequency modulations.

### Consequences for social interactions

In an environment as crowded and noisy as a little auk colony, finding and communicating with one’s partner or other members of one’s social network may be particularly challenging. Yet little auks successfully maintain partnership over the years^31^, coordinate parental care^26^, and find each other not only after foraging trips, but also upon returning from annual migrations. The extent to which vocalisations facilitate these different aspects of the species’ social life remains an open question, but the elaborate vocal communication system clearly suggests that acoustic cues are of importance.

While nothing is known about the social networks of the little auk outside of the breeding context, it seems possible that extra-pair or non-breeding relationships may occur, as is also suggested by the complex vocal communication within the colony. In fact, most of the described call types are social vocalisations related to a wide range of social interactions outside the breeding couple, from predator warning to, possibly, nest protection. Additionally, changes to the acoustic structure of the calls corresponding to behavioural and affective contexts can inform conspecifics about the potential risks and opportunities, increasing the fitness of the whole colony.

Some of the observed variance in acoustic parameters within call types and contexts may be due to individual differences. For example, the *classic* call, which is a long call comprised of three types of harmonic syllables, seems to hold great potential for coding information about the individual. While we were not able to establish the identity of callers in this study, it seems plausible that this call type may be used as a vocal signature, enabling the little auks to find their partners and neighbours after migrations and gull attacks, but also perhaps to coordinate other behaviours. Dedicated studies with known individuals are necessary to assess the level of individual vocal stereotypy in this species.

Finally, changes in acoustic parameters can be related to other, physiological factors, such as side-effects of amplitude and/or frequency modulation in noisy environments^43,44^. Since different factors can contribute to the overall acoustic structure of a vocalisation, further investigations controlling for their impact would help us disentangle the physiological vs. behavioural effects on the vocalisation structure.

### Caveats and issues

Working on seabird bioacoustics comes with a number of challenges. While as a species the little auk is very vocal, individual animals often vocalise at unpredictable times and places. For this reason, and to ensure that we captured the whole spectrum of calls produced in the colony over a longer period, this study used passive acoustic recording of the colony, and therefore we cannot make any assumptions about how sex, age, size, and identity of the vocalising animal influence vocal behaviour of the species.

While the sample size used in this study is not very large—and in the case of valence analysis was narrowed down to calls which could be classified as *positive* or *negative* with high probability, so e.g. excluding social interactions with non-partners—it is the feasible output of long-term monitoring efforts of the study species. Further data—ideally, directly recorded vocalisations of known, positive and negative valence within one call type—would be beneficial, and enable us to make more final conclusions. Nevertheless, we believe that this unique dataset is a valuable contribution and can form a basis for future studies, indicating potential for vocal expression of emotions in an understudied but important group of birds.

Using audio (and possibly video) recording devices mounted on individuals could resolve some of these issues. However, while these prove useful on larger species^7^, little auks are small and likely to be sensitive to such a burden^45^. For this reason, it is for the moment impossible to describe the vocal behaviour of the little auks during foraging trips or migrations. Nevertheless, we are confident we managed to capture the whole spectrum of vocalisations produced by the little auk at the colony over mating and incubation.

### Conclusion

This study identifies and provides a quantified description of eight call types produced by adult little auks over mating and incubation, setting a framework for future studies of the vocal behaviour of this species. It also suggests emotional effects on the acoustic parameters of these calls, such as fundamental frequency, duration, and spectral centre of gravity. This is the first time that vocal expression of affective states has been studied in a seabird, shining a new light on avian behaviour. Due to the technical limitations of this study, its results should be taken with precautions, serving only as an indicator that such effects might indeed exist in this group.

## Methods

### Ethics and permits

Fieldwork was performed under permission from the Governor of Svalbard (17/00663-13, 20/00373-2, 20/00373-8).

### Study site and recording set-up

All data were collected in the little auk colony in Hornsund, Spitsbergen (77° 00′ N, 15° 33′ E), one of Svalbard’s biggest breeding aggregations of the species^29^. Recordings were made over the breeding seasons (June–August 2019–2021), during mating and incubation periods. These periods were chosen to ensure only adult birds were present in the colony.

Audio material was collected via an Olympus ME-51S stereo microphone (frequency response 100–15,000 Hz) placed right outside (mating) or inside (incubation) individual nests (n = 30) in such a way as to not disturb the birds’ normal activities. Each microphone was connected to an Olympus LS-3 or LS-P4 digital voice recorder (sampling rate 48 kHz, 16 bits) placed outside of the nest and hidden under a rock.

Recording sessions took place for several hours during different stages of mating (three sessions per nest) and incubation (three sessions per nest) periods, recording vocalisations of the focal nest owners, their mutual interactions, interactions with neighbours, as well as all other vocalizations produced in the nest vicinity, i.e. neighbouring pairs and flocks of birds circling above the colony plot. All recording sessions were equal in duration, and spaced equally in time for all the monitored nests.

Additionally, recordings were made during handling the birds for standard ornithological procedures (while ringing, weighting) via a hand-held recorder (Olympus LS-12) with a built-in microphone. Although adult little auks are often silent during handling, we managed to record handling vocalisations of 21 individuals.

### Sound selection

We manually processed a total of 508 h 27 min of recordings, extracting all clear calls found within this set. These calls were grouped into call types based on visual inspection of the spectrograms (Hann window, FFT-length = 715) and the associated production contexts of the calls. From the extracted calls, we selected 30 high-quality (i.e. non-overlapping, untrimmed calls with the best available signal-to-noise ratio) calls per each identified call type, i.e. a total of 240 calls.

While it was not possible to assign specific calls to particular individuals, owing to the sampling design (i.e., recoding at multiple nest locations) and further sound selection routine (i.e., avoiding sampling from the same nest or vocalisation bout) we are confident that most of the selected audio samples originated from different individuals, as so could be treated as independent data points in further analyses. In the case of the *handing call*, with only 21 individuals recorded, we were forced to sample nine individuals twice—these individuals were chosen based on the signal quality, i.e. recordings with the best signal-to-noise ratio were selected.

### Context and valence attribution

The context of vocal attribution was assessed based on previous behavioural observations using expert knowledge approach, matching the observed call types to their typically associated production contexts. This was based on direct long-term observations in the field (ad libitum observations of focal animals and colony scanning), analyses of focal video recordings from 2019 to 2020, and previous literature^33^.

Valence is one of the main dimensions of emotions^17,19^. The valence of a context can be assumed based on threats/promoters of fitness—that means, contexts that are related to certain adverse or beneficial situations^17,19,46^. Therefore, contexts that threaten fitness and would normally be avoided were assumed to trigger negative states, while those that promote fitness and should be approached were assumed to elicit positive states^19,46,47^ (Table 1). Little auks parents take care of their brood in a coordinated^26^ manner where both partners contribute equally^26,31^, with no parental conflict observed. Thus, we assumed that interactions with the social partner are predicted to be positive, interactions with a predator or human (i.e. handling) should be negative, interactions with other birds in the vicinity of own nest are likely negative, and other contexts are of unknown valence. For further analysis, we selected only the calls that fell into the *positive* or *negative* categories. This was done to avoid confusion in situations where the behavioural context/meaning is not always clear, such as communication with birds other than nesting partners (*likely negative*).

### Sound analysis

Calls were analysed in Praat software^48^ using a custom built script adjusted to the little auk^22,49,50^ (Supplementary Text 1), extracting a set of 20 acoustic parameters (specified in Supplementary Table 2).

### Statistical analysis

#### Repertoire

All analyses were performed in R environment (v. 4.1.3)^51^. Summary statistics of standard acoustic variables (Supplementary Table 2) were calculated for each call type. Principal component analysis (PCA) was performed (using *stats* package^52^, function *prcomp*) on all acoustic parameters to reduce data dimensions and cross-check call types’ classification. This was followed by Discriminant Function Analysis (DFA; *MASS* package^53^, function *lda*) using PC scores with eigenvalues > 1. The correct classification rate obtained from the DFA with leave-one-out cross-validation was then compared to 1000 chance levels calculated by applying a randomisation procedure (permutation test), in order to investigate if call types were acoustically distinct from each other.

#### Vocal expression of affective valence

The first five PCA dimensions had eigenvalues > 1 (Kaiser’s criterion; Supplementary Table 3). Based on the raw variables’ contribution to these dimensions (i.e., over 6% contribution to all dimensions, according to the scree plot cut-off; Supplementary Table 4), we selected the following acoustic parameters for subsequent tests: Max *f0*, Range *f0*, Mean *f0*, Q50%, *f0* Abs Slope, *f0* var, End *f0*, and Sound duration (Supplementary Table 2).

Since many of the raw acoustic parameters we analysed are correlated (e.g. the different aspects of fundamental frequency changes), we have decided to use a multivariate analysis of variance based on the PC scores. To investigate variation at the level of the whole call structure, the first five PCs were entered as response variables into a MANOVA (*stats* package^52^, *manova* function). The MANOVA included the assumed valence of the situation (positive or negative, Table 1) as explanatory variable and call types as a fixed factor to control for its effect.

Additionally, to understand the specific direction of changes in the eight selected parameters, their raw values were entered as outcome variables into linear models (LM; *stats* package^52^, *lm* function; 8 models in total) including valence (Table 1; calls of *possibly negative* and *unknown* valence were not included in the analysis but visualized on the plots (Supplementary Figs. 1–8) as a fixed factor).

## Supplementary Information

Supplementary Legends.

Supplementary Figure 1.

Supplementary Figure 2.

Supplementary Figure 3.

Supplementary Figure 4.

Supplementary Figure 5.

Supplementary Figure 6.

Supplementary Figure 7.

Supplementary Figure 8.

Supplementary Table 1.

Supplementary Table 2.

Supplementary Table 3.

Supplementary Table 4.

Supplementary Information.

## Data Availability

Call samples with their respective spectrograms, as well as the raw data generated in this study are available at https://osf.io/83fx6/?view_only=bd9336e27dfe4f93a0e9338ccc016597.
