# Supplementary material for: Seabird’s cry: repertoire and vocal expression of contextual valence in the little auk (Alle alle)
Source: Sci Rep. 2023 May 27;13:8623. doi: 10.1038/s41598-023-35857-3 (PMC10224962; doi:10.1038/s41598-023-35857-3)
Supplement: Supplementary file 1 — Supplementary Legends. [file 41598_2023_35857_MOESM1_ESM.docx]

**Supplementary Material legends**

**Supplementary Table 1.** Model results: MANOVA for assigned valence.

**Supplementary Table 2.** Acoustic parameters extracted for each vocalisation (adapted from Briefer *et al.* 2017). Whether each parameter was selected for statistical analysis based on PCA results and in summary statistics of repertoire descriptions is indicated.

**Supplementary Table 3.** PCA results: eigenvalues and percentage of variance for 17 dimensions.

**Supplementary Table 4.** Raw variables’ contributions (%) to the first five PCA dimensions. Variables in bold were selected for MANOVA.

**Supplementary Figure 1.** Effect of the assigned valence on the maximum *f0*. *Negative* and *positive* categories are included in the analyses, while *possibly negative* and *unknown* categories are only plotted for comparison.

**Supplementary Figure 2.** Effect of the assigned valence on the mean *f0*. *Negative* and *positive* categories are included in the analyses, while *possibly negative* and *unknown* categories are only plotted for comparison.

**Supplementary Figure 3.** Effect of the assigned valence on the *f0* range. *Negative* and *positive* categories are included in the analyses, while *possibly negative* and *unknown* categories are only plotted for comparison.

**Supplementary Figure 4.** Effect of the assigned valence on Q50%. *Negative* and *positive* categories are included in the analyses, while *possibly negative* and *unknown* categories are only plotted for comparison.

**Supplementary Figure 5.** Effect of the assigned valence on *f0* Abs Slope. *Negative* and *positive* categories are included in the analyses, while *possibly negative* and *unknown* categories are only plotted for comparison.

**Supplementary Figure 6.** Effect of the assigned valence on *f0* var. *Negative* and *positive* categories are included in the analyses, while *possibly negative* and *unknown* categories are only plotted for comparison.

**Supplementary Figure 7.** Effect of the assigned valence on the End *f0*. *Negative* and *positive* categories are included in the analyses, while *possibly negative* and *unknown* categories are only plotted for comparison.

**Supplementary Figure 8.** Effect of the assigned valence on sound duration. *Negative* and *positive* categories are included in the analyses, while *possibly negative* and *unknown* categories are only plotted for comparison.
