# Supplementary figures and images for: Seabird’s cry: repertoire and vocal expression of contextual valence in the little auk (Alle alle)
Source: Sci Rep. 2023 May 27;13:8623. doi: 10.1038/s41598-023-35857-3 (PMC10224962; doi:10.1038/s41598-023-35857-3)

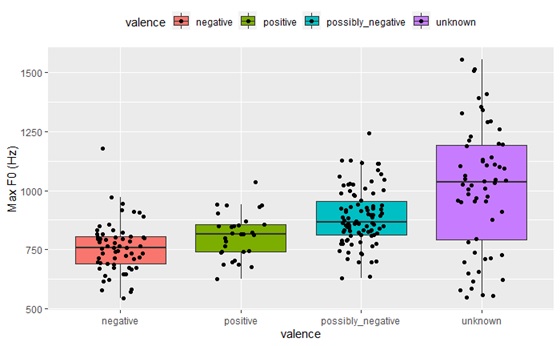

Supplement: Supplementary file 2 — Supplementary Figure 1. [file 41598_2023_35857_MOESM2_ESM.jpg]

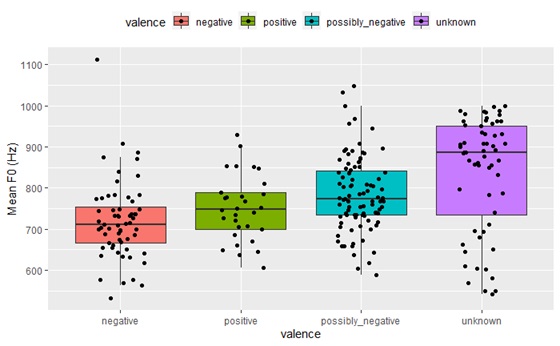

Supplement: Supplementary file 3 — Supplementary Figure 2. [file 41598_2023_35857_MOESM3_ESM.jpg]

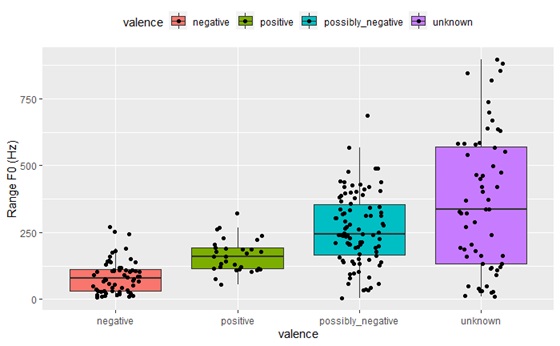

Supplement: Supplementary file 4 — Supplementary Figure 3. [file 41598_2023_35857_MOESM4_ESM.jpg]

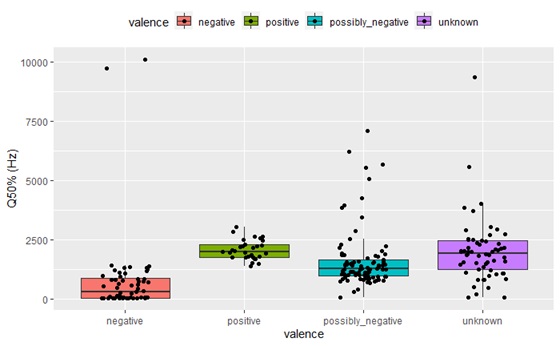

Supplement: Supplementary file 5 — Supplementary Figure 4. [file 41598_2023_35857_MOESM5_ESM.jpg]

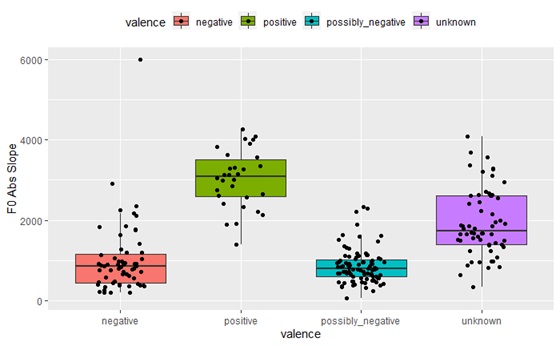

Supplement: Supplementary file 6 — Supplementary Figure 5. [file 41598_2023_35857_MOESM6_ESM.jpg]

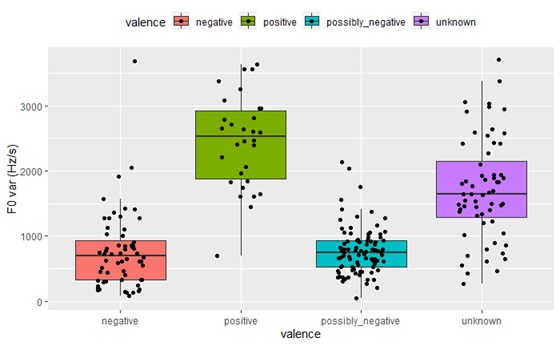

Supplement: Supplementary file 7 — Supplementary Figure 6. [file 41598_2023_35857_MOESM7_ESM.jpg]

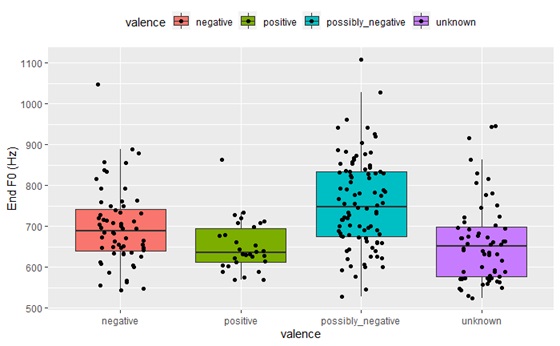

Supplement: Supplementary file 8 — Supplementary Figure 7. [file 41598_2023_35857_MOESM8_ESM.jpg]

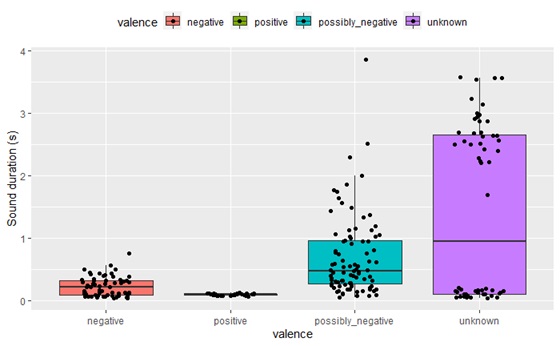

Supplement: Supplementary file 9 — Supplementary Figure 8. [file 41598_2023_35857_MOESM9_ESM.jpg]
