# Supplementary material for: Seabird’s cry: repertoire and vocal expression of contextual valence in the little auk (Alle alle)
Source: Sci Rep. 2023 May 27;13:8623. doi: 10.1038/s41598-023-35857-3 (PMC10224962; doi:10.1038/s41598-023-35857-3)
Supplement: Supplementary file 10 — Supplementary Table 1. [file 41598_2023_35857_MOESM10_ESM.docx]

**Supplementary Table 1.** Model results: MANOVA for assigned emotional valence.

|  | **Df** | **Pillai** | **Approx. F** | **Num Df** | **Den Df** | **Pr(>F)** |
| --- | --- | --- | --- | --- | --- | --- |
| **valence** | 1 | 0.75 | 48.93 | 5 | 83 | < 0.001 |
| **call type** | 1 | 0.74 | 47.02 | 5 | 83 | < 0.001 |
| **Residuals** | 87 |  |  |  |  |  |
