# Supplementary material for: Seabird’s cry: repertoire and vocal expression of contextual valence in the little auk (Alle alle)
Source: Sci Rep. 2023 May 27;13:8623. doi: 10.1038/s41598-023-35857-3 (PMC10224962; doi:10.1038/s41598-023-35857-3)
Supplement: Supplementary file 11 — Supplementary Table 2. [file 41598_2023_35857_MOESM11_ESM.docx]

**Supplementary Table 2.** Acoustic parameters extracted for each vocalisation (adapted from Briefer *et al.* 2017). Whether each parameter was selected for statistical analysis based on PCA results and in summary statistics of repertoire descriptions is indicated.

| **Abbreviation** | **Description** | **Analysis** | **Repertoire** |
| --- | --- | --- | --- |
| ***f*0 Mean (Hz)** | Mean fundamental frequency value across the vocalisation | + | + |
| ***f*0 Start (Hz)** | Fundamental frequency value at the start of the vocalisation | - | - |
| ***f*0 End (Hz)** | Fundamental frequency value at the end of the vocalisation | + | - |
| ***f*0 Max (Hz)** | Maximum value of the fundamental frequency across the vocalisation | + | + |
| ***f*0 Min (Hz)** | Minimum value of the fundamental frequency across the vocalisation | + | + |
| ***f*0 Range** | Range of the fundamental frequency frequency across the vocalisation | + | - |
| **Time *f*0 Max (%)** | Percentage of time when the maximum F0 frequency occurs within the vocalisation | - | - |
| ***f*0 Abs Slope** | Absolute slope of F0 frequency | + | - |
| ***f*0 Var (Hz/s)** | Cumulative variation in F0 frequency divided by the total vocalisation duration | + | - |
| ***f*M Rate (s^-1^)** | Frequency modulation rate | - | + |
| ***f*M Extent (Hz)** | Mean peak-to-peak variation of each frequency modulation | Excluded (some missing values) | Excluded (some missing values) |
| **Q25% (Hz)** | Frequency value at the upper limit of the first quartiles of energy | - | + |
| **Q50% (Hz)** | Frequency value at the upper limit of the second quartiles of energy | + | + |
| **Q75% (Hz)** | Frequency value at the upper limit of the third quartiles of energy | - | + |
| ***f*peak (Hz)** | Peak frequency | - | - |
| **Dur (s)** | Duration of the vocalisation | + | + |
| **AM Var (dB/s)** | Cumulative variation in amplitude divided by the total vocalisation duration | - | - |
| **AM Rate (s^-1^)** | Amplitude modulation rate | - | + |
| **AM Extent (dB)** | Mean peak-to-peak variation of each amplitude modulation | Excluded (some missing values) | Excluded (some missing values) |
| **Harm (dB)** | Harmonicity (Harmonics-to-Noise Ratio) | Excluded (some missing values) | Excluded (some missing values) |
