# Supplementary material for: Seabird’s cry: repertoire and vocal expression of contextual valence in the little auk (Alle alle)
Source: Sci Rep. 2023 May 27;13:8623. doi: 10.1038/s41598-023-35857-3 (PMC10224962; doi:10.1038/s41598-023-35857-3)
Supplement: Supplementary file 12 — Supplementary Table 3. [file 41598_2023_35857_MOESM12_ESM.docx]

**Supplementary Table 3.** PCA results: eigenvalues and percentage of variance for 17 dimensions.

|  | **eigenvalue** | **percentage of variance** | **cumulative percentage of variance** |
| --- | --- | --- | --- |
| **comp 1** | 4.82 | 28.36 | 28.36 |
| **comp 2** | 2.71 | 15.95 | 44.31 |
| **comp 3** | 2.46 | 14.47 | 58.78 |
| **comp 4** | 1.95 | 11.50 | 70.28 |
| **comp 5** | 1.32 | 7.780 | 78.06 |
| **comp 6** | 0.85 | 5.02 | 83.08 |
| **comp 7** | 0.74 | 4.36 | 87.43 |
| **comp 8** | 0.54 | 3.18 | 90.61 |
| **comp 9** | 0.49 | 2.88 | 93.48 |
| **comp 10** | 0.34 | 2.00 | 95.49 |
| **comp 11** | 0.32 | 1.85 | 97.34 |
| **comp 12** | 0.15 | 0.90 | 98.24 |
| **comp 13** | 0.13 | 0.75 | 98.99 |
| **comp 14** | 0.09 | 0.55 | 99.54 |
| **comp 15** | 0.06 | 0.32 | 99.86 |
| **comp 16** | 0.02 | 0.14 | 100.00 |
| **comp 17** | 0.00 | 0.00 | 100.00 |
