# Supplementary material for: Seabird’s cry: repertoire and vocal expression of contextual valence in the little auk (Alle alle)
Source: Sci Rep. 2023 May 27;13:8623. doi: 10.1038/s41598-023-35857-3 (PMC10224962; doi:10.1038/s41598-023-35857-3)
Supplement: Supplementary file 13 — Supplementary Table 4. [file 41598_2023_35857_MOESM13_ESM.docx]

**Supplementary Table 4.** Raw variables’ contributions (%) to the first five PCA dimensions. Variables in bold were selected for MANOVA.

| **Variable** | **Dim.1** | **Dim.2** | **Dim.3** | **Dim.4** | **Dim.5** |
| --- | --- | --- | --- | --- | --- |
| **Mean *f0* (Hz)** | 14.41 | 2.27 | 7.01 | 0.03 | 0.10 |
| Start *f0* (Hz) | 6.55 | 9.16 | 1.32 | 0.63 | 12.97 |
| **End *f0* (Hz)** | 0.11 | 2.52 | 27.08 | 5.21 | 1.99 |
| **Max *f0* (Hz)** | 17.73 | 0.09 | 2.54 | 1.88 | 0.03 |
| Min F0 (Hz) | 0.44 | 14.43 | 11.63 | 6.48 | 7.84 |
| **Range *f0* (Hz)** | 15.32 | 1.85 | 0.01 | 6.16 | 1.99 |
| Time max F0 (%) | 2.05 | 0.43 | 8.00 | 5.08 | 22.28 |
| ***f0* Abs Slope** | 3.48 | 14.73 | 10.50 | 0.02 | 5.27 |
| ***f0* var (Hz/s)** | 5.55 | 12.29 | 8.96 | 0.03 | 5.00 |
| *f*M Rate (s-1) | 0.63 | 0.25 | 0.96 | 2.00 | 31.20 |
| Q25% (Hz) | 8.74 | 0.09 | 3.88 | 1.56 | 3.77 |
| **Q50% (Hz)** | 6.07 | 5.76 | 3.38 | 19.13 | 0.09 |
| Q75% (Hz) | 1.76 | 9.67 | 0.33 | 20.11 | 0.13 |
| *f*peak (Hz) | 2.64 | 1.84 | 3.85 | 12.46 | 2.66 |
| **Sound duration (s)** | 10.77 | 6.17 | 0.02 | 5.14 | 2.61 |
| AM var (dB/s) | 2.31 | 5.89 | 4.52 | 13.56 | 1.95 |
| AM rate (s-1) | 1.42 | 12.57 | 6.01 | 0.49 | 0.11 |
