# Supplementary material for: Seabird’s cry: repertoire and vocal expression of contextual valence in the little auk (Alle alle)
Source: Sci Rep. 2023 May 27;13:8623. doi: 10.1038/s41598-023-35857-3 (PMC10224962; doi:10.1038/s41598-023-35857-3)
Supplement: Supplementary file 14 — Supplementary Information. [file 41598_2023_35857_MOESM14_ESM.docx]

**Supplementary Text 1**

**Praat Settings**

Here, we provide a detailed description of the acoustic analysis performed in Praat software using a custom-made script^46–48^. Settings used to extract the 20 acoustic parameters presented in Supplementary Table 1 are described below (Praat commands indicated in brackets; see Supplementary Table 1 for abbreviations of the parameters used).

1. **Duration**. The duration (Dur) was measured as the total duration of each wav file (s), corresponding to individual calls manually extracted from the recordings, based on the visualisation of both the oscillogram and spectrogram.

2. **Amplitude modulation**. AM Var, AM Rate, and AM Extent were calculated from the intensity contour of each individual call, using the [Sound: To Intensity] command (minimum pitch = 500 Hz, time step = 0.005 s)^54^.

3. **Source-related acoustic features**. *f0* contour or each call was extracted using a cross-correlation method ([Sound: To pitch (cc)] command; time step = 0.005 s, pitch floor = 500 Hz, pitch ceiling = 2000 Hz). We included the following *f0* frequency values: *f0* at the start (*f0* Start) and at the end (*f0* End); the mean (*f0* Mean), minimum (*f0* Min) and maximum (*f0* Max); percentage of time when the maximum *f0* frequency occurs within the vocalisation (Time *f0* Max); the *f0* mean absolute slope (*f0* Abs Slope); and the *f0* range (*f0* Range). To characterise *f0* variation along the call, we measured the mean *f0* variation per second (*f0* Var) calculated as the cumulative variation in the *f0* contour in Hertz divided by call duration. Finally, we measured the number of complete cycles of *f0* modulation per second (*f*M Rate) and the mean peak-to-peak variation of each *f0* modulation (*f*M Extent)^52^.

4. **Spectrum-related parameters.** Q25%, Q50%, and Q75% were measured on a spectrum applied to the whole call, and *f*Peak was measured on a cepstral-smoothed spectrum (command [Create: Cepstral smoothing]; bandwidth = 100 Hz).

5. **Noise**. Harmonicity (Harm) was measured using the [Sound: To Harmonicity (cc)] command (time step = 0.005 s, minimum pitch = 500 Hz, silence threshold = 0.2, periods per window = 1).

*f*M Extent (Hz), AM Extent (dB) and Harm (dB) could not be extracted from some of the calls. All other parameters could be measured in all calls.

Praat spectrogram settings: view range max: 8000; window length: 0.008; dynamic range: 60.
